# Dent Disease Type 1: A Diagnostic Dilemma and Review

**DOI:** 10.7759/cureus.23910

**Published:** 2022-04-07

**Authors:** Ryan B Soares, Naina Bhat

**Affiliations:** 1 Internal Medicine, Dr. Roque Ferreira's Memorial Hospital, Margao, IND; 2 Pediatrics, St. John's Medical College Hospital, Bengaluru, IND

**Keywords:** x-linked, gh therapy, renal failure, lmwp, tubulopathy, hypophosphatemic rickets, dent disease

## Abstract

This case report describes a boy with a rare genetic disease that primarily affects the kidneys and has implications on growth and development. Dent disease type 1 is an X-linked tubulopathy mainly caused by inactivating mutations in the chloride voltage-gated channel 5 (CLCN5) gene. It is a rare but important diagnosis for children with variable phenotypic presentations that can include low molecular weight proteinuria (LMWP), nephrocalcinosis, bony deformities and possible progression to early-onset renal failure. A delay in diagnosis is often encountered when it comes to Dent disease. This is due to the similarities in presentation of the disease to other commonly seen pediatric conditions (such as minimal change nephrotic syndrome, nutritional rickets, renal tubular acidosis [RTA], etc.) and also since it can present with variable phenotypes and has a great amount of allelic heterogeneity. In this case, it was diagnosed after 13 years from symptom onset. The patient was subjected to alternative forms of medicine, multiple working diagnoses and associated treatments at various hospitals which most likely contributed to a faster disease progression. In addition to the treatment of the disease, growth hormone (GH) therapy has proven to be beneficial but was not offered to this patient. In this case, we would also like to report some rare findings such as persistent hypercholesterolemia and steroid-resistant nephrotic syndrome (SRNS) biopsy pattern. We decided to pursue this particular disease to highlight the importance of having a high clinical suspicion with a view to attain a definitive diagnosis and instituting appropriate treatment as soon as possible. We also highlight the importance of keeping the patient informed about their disease, the possible therapeutic options and the importance of genetic counselling and patient education.

## Introduction

Dent disease type 1 is an X-linked recessive renal tubular disease that classically manifests with low molecular weight proteinuria (LMWP), hypercalciuria leading to nephrocalcinosis/nephrolithiasis and one or more additional features like hypophosphatemia, hematuria, bony deformities, short stature and eventually, progression to chronic kidney disease (CKD). The incidence of low molecular weight proteinuria in both Dent disease type 1 and type 2 populations is 100%. However, the incidence of other manifestations is not high, which is similar to previously reported data. Therefore, LMWP is a key clinical feature that should alert pediatricians to the possibility of Dent disease [1]. Loss-of-function mutations of chloride channel 5 (CLC-5), a chloride/proton exchanger on renal endosomes of proximal tubular cells, and to a lesser extent in cells of the medullary thick ascending limb and the intercalated cells of the collecting duct [2], encoded by the chloride voltage-gated channel 5 (CLCN5) gene located on chromosome Xp11.22, results in Dent disease (Online Mendelian Inheritance in Man [OMIM]:300009). Males younger than 10 years of age may manifest only low molecular weight proteinuria and/or hypercalciuria and they are usually asymptomatic. Patients with Dent disease type 2 (Lowe syndrome) harbour inactivating mutations of the inositol polyphosphate-5-phosphatase gene (OCRL) (Online Mendelian Inheritance in Man [OMIM]:300355). They may additionally also have cataracts, mild intellectual disability and/or elevated muscle enzymes.

## Case presentation

A 15-year and eight-month-old male presented to the clinic with burning micturition, fever and suprapubic pain for four days. This was his third episode of a lower urinary tract infection in six months. On evaluation, his blood pressure was 110/62 millimetres of mercury (mmHg) and a peripheral blood oxygen saturation of 99% on room air. Anthropometric measurements included a weight of 26.1kg (<3rd centile for age), a height of 125.1cm (<3rd centile), and a body mass index (BMI) of 15.9kg/m^2 ^(10th to 25th centile). On assessing the parents' height, the mother was 152.5cm while the father was 164.0cm. This gave a mid-parental height (MPH) value of 164.75cm, which was between the 15th to 50th centile. A sexual maturity rating (SMR) score as per the Tanner and Whitehouse classification system was Ph1, G2. Testicular volume was 3cc and 4cc on the right and left, respectively, with a stretched penile length (SPL) of 4.0cm. Other tell-tale signs of puberty such as growth spurt, acne vulgaris, behavioural changes, deepening of voice etc. were not present in this patient. Visual acuity was 6/6 in both eyes, with normal near and colour vision. Due to this peculiar clinical presentation, we were curious and wanted to know more about the patient’s history. Diagnostic tests ordered at this point were as follows: biochemical analysis was normal - sodium (134mEq/l), potassium (3.9mEq/l), chloride (99mEq/l), calcium (9.7mg/ dl), alkaline phosphatase (295 IU/l, normal 50-440) and acid-base balance (pH 7.420, bicarbonate 23.3mEq/l); serum total protein and albumin were 8.0g/dl and 4.9g/dl, respectively; vitamin D levels were 27.6ng/ml (deficiency: <30ng/ml), while the cholesterol was elevated (373mg/dl). Renal function tests were abnormal (serum creatinine [1.8mg/dl], urea [49.78mg/dl]). Urinary spot protein was 3+. An estimated glomerular filtration rate (eGFR) of 58ml/min/1.73m^2^ and a 24hr albuminuria of 28.3mg/g placed the patient in stage G3a A1 based on the Kidney Disease Improving Global Outcomes (KDIGO) chronic kidney disease guidelines [3]. A hormone panel showed a testosterone level of 0.2ng/ml (normal 0.47-9.8ng/ml for his age), LH 1.45mIU/ml (normal 1-3.7mIU/ml), FSH 1.08mIU/ml (normal 1.0-8.6mIU/ml) and a normal thyroid profile. A radiograph of the wrist was obtained, and bone age of 10.1 to 11.4 years was determined.

Past history

The patient is the second child born to non-consanguineous parents. He has two sisters. He was delivered via vaginal delivery after an uneventful pregnancy with a birth weight recorded as 4000 grams. The child was exclusively breastfed for six months, following which he was started on a semi-solid diet. The child was feeding well and gaining weight, with good urine output. Immunizations were up to date. At 18 months of age, the child started passing frothy urine and refused to feed. Subsequently, he started losing weight and was then evaluated at a tertiary care centre. On evaluation, vital signs were within normal limits with a blood pressure of 86/52mmHg. His weight at the time was 8.6kg (<3rd centile) and his length was 80.0cm (15th to 50th centile). A urinalysis was positive for nephrotic parameters. The boy was diagnosed to have idiopathic nephrotic syndrome and started on oral prednisolone at a dose of 60mg/m^2^ for six weeks. The patient also received three pulses of intravenous methylprednisolone (500mg/m^2^) in view of persistent proteinuria with an elevated urine protein creatinine ratio (UPC) but there was still no improvement. An immunology panel showed normal serum complement levels and negative anti-nuclear antibodies (ANA). An ultrasound of the kidneys revealed no abnormalities. Hence, a renal biopsy was done and reported as mild mesangial hypercellularity with matrix expansion with no areas of sclerosis. A provisional diagnosis of minimal change nephrotic syndrome (MCNS) was made. However, other typical signs of MCNS such as hypocomplementemia and edema were lacking in this patient. The child was restarted on oral steroids and was lost to follow-up for three years. After this, the child presented with bowing of the lower limbs (genu valgum) and was diagnosed to have nutritional rickets. He was given mega doses of vitamin D [600,000 U stat, oral calcium and vitamin D supplements and 300,000 + 300,000 U vitamin D injections five months apart due to persistent limb deformities. At this point, the proteinuria continued (UPC 9.36). Due to the lack of improvement, the patient did not follow up for another year.

During this period, the child was continued on steroids for around one to two months, with variable adherence to daily dosing. The parents then sought alternative and indigenous forms of medicine, but the symptoms worsened, and the child was not gaining weight and was severely stunted. This prompted the parents to seek medical care once more and the child now presented with severe bowing of the legs, vomiting, irritability, polyuria and abdominal pain. This was attributed to vitamin D intoxication. Renal ultrasound findings were suggestive of early medullary nephrocalcinosis. Due to persistent hypophosphatemia, proteinuria and occasional hypokalemic episodes, a tubular pathology was sought. The patient was told to have steroid-resistant nephrotic syndrome (SRNS). However, another trial of steroids for two + four + two weeks was still given, which only pushed the patient into steroid toxicity and he presented after eight weeks with cushingoid facies, elevated blood pressure and an irritable mood with deranged laboratory parameters. A second renal biopsy was done in which 28 glomeruli were studied, of which six were obsolescent with segmental sclerosis. The vessels showed hyperplasia of the tunica media. No crescents or proliferative/necrotizing lesions were seen. The immunofluorescence (IF) of the specimen was negative for immunoglobulins and complement proteins. The biopsy was reported as focal segmental glomerulosclerosis (FSGS) and as a last resort, the patient was started on oral tacrolimus therapy. Due to a lack of response, he was referred to a hospital outside the state. Here, a thorough evaluation was done. The patient had a blood pressure of 94/60mmHg. His weight was 18kg (3rd centile) and his height was 115.0cm (3rd to 15th centile). Systemic examination was normal, with genu valgum deformity (intermalleolar distance [IMD] 11cm). Laboratory investigations were as follows: biochemical analysis showed normal levels of sodium (137mEq/l), potassium (3.6mEq/l, normal 3.0-5.0) and calcium (8.9mg/ dl), alkaline phosphatase (579 IU/l, normal 50-440), acid-base balance (pH 7.391, bicarbonate 27mEq/l) and normal creatinine clearance (97ml/min/1.73 m2). Serum cholesterol was 220mg/dl with normal LDL, HDL and triglyceride levels. However, he had proteinuria (91.0mg/dl spot protein; 1350mg/l/day microalbuminuria [normal 0-50mg/l/day]), UPC 5.3 (normal <0.20), hypercalciuria (24-h urinary calcium - 213mg/day [12.5mg/kg/day, normal <4.0mg/kg/day], calcium creatinine ratio 0.6 (normal <0.2) and serum PTH 100pg/ml (normal 10.0-69.0pg/ml). Based on this and to evaluate for tubular phosphaturia, tubular maximum phosphate reabsorption per glomerular filtration rate (TmP/GFR) was done and it was 2.3mg/dl, (normal for a five to 12-year-old boy is 2.8 - 4.4mg/dl). A renal ultrasound now showed bilateral medullary nephrocalcinosis with normal-sized kidneys. Because of normal serum albumin levels, no edema and past medical records, nephrotic syndrome was not considered and a diagnosis of hypophosphatemic rickets was made. He was started on oral potassium, phosphate, angiotensin-converting enzyme (ACE) inhibitors and atorvastatin with cautious use of thiazide diuretics. Genetic testing, growth hormone and insulin-like growth factor 1 (IGF-1) levels were not done.

The patient was then followed up every three months and his laboratory values were monitored. However, frequent hypokalemic and hypophosphatemic episodes with poor cholesterol control were noticed. This was attributed to poor compliance and inadequate dose titration. A series of repeat renal ultrasounds yearly showed multiple bilateral calcific foci with right-sided hydronephrosis and proximal hydroureter. During this time, the child was gradually clinically deteriorating, with frequent urinary tract infections and a rising creatinine value. A corrective osteotomy was done for the genu valgum with good results. Finally, at age 14, exome sequencing was ordered to establish a definitive molecular diagnosis and a hemizygous missense mutation p.Ser244Leu (c.731C>T) in exon 7 of the CLCN5 gene was identified which corresponds to a diagnosis of Dent disease type 1. A summary of laboratory tests of our patient over a period of 13 to 14 years is illustrated in Table 1. 

**Table 1:** Laboratory and urinary analytes of our patient over a 13 to 14 year period Cr: Creatinine, Ca: Calcium, P: Phosphate, Mg: Magnesium, Na: Sodium, K: Potassium, Cl: Chloride, HCO_3 _: Bicarbonate, pH: Potential of Hydrogen, PTH: Parathyroid hormone, ALP: Alkaline phosphatase, TG: Triglyceride, UPC: Urine protein creatinine ratio, eGFR: Estimated glomerular filtration rate

Age (in years)	Reference range	1.5	4	5	6	7	8	9	10	12	14	15
SERUM												
Cr (mg/dl)	0.6 – 1.1	0.6	0.6	0.6	0.6	0.5	0.8	0.8	0.9	1.0	1.2	1.8
Total protein/Albumin (g/L)	6.0 – 8.3/3.4 – 5.4				7.3/4	7.2/4	7.0/3.9	7.8/4			7.2/4.7	8.0/4.9
Ca (mg/dl)	8.6 – 10.3	9.9	10	9.8	10.1	8.9	8.8	9.7	10	10.2	9.8	9.6
P (mg/dl)	2.8 – 4.5	3.5	3.6		2.4	2.1	3.7	1.7		2.3	3.8	2.7
Mg (mg/dl)	1.3 – 2.1				3.1	2.7		1.64	2.2	1.6		
Na (mEq/l)	135 – 145				136	137	130	128	133	135	137	130
K (mEq/l)	3.5 – 5.2			3.5	3.9	3.6	3.2	3.6	4.0	2.62	3.1	3.1
Cl (mEq/l)	96 – 106				112	100	98	94	104	100	91	90
HCO_3 _(mmol/l)	22 - 28			22.9	21.4	27.0	22.8				19.5	29.4
pH	7.35 – 7.45			7.36		7.41	7.48				7.43	7.52
PTH (pg/ml)	10.0 – 69.0		2.5	3.5	3.3	100						
Vitamin D (ng/ml)	>30		56		77					15		13.3
ALP (U/l)	44 - 147		608		640	579	508	297		425	534	304
Cholesterol/TG (mg/dl)	<200/<150	188			191	220	342/74	212/109	322	260/87	230/111	306/74
URINE												
Protein/Albumin	-	(4+)	(1+)	(4+)	(3+)	(2+)	(2+)	(2+)	(2+)	(2+)	(2+)	(2+)
Occult blood	-	-	-	8-10		4-5	-	-	-	-	-	-
24 h protein (mg/m^2^/hr)	<4.0				78.7	74.8						49.0
24 h calcium (mg/kg/day)	<4.0			22.9	18.2	12.5					15.7	
UPC	<0.2	10	3.71		3.46	5.3	2.11	1.05	2.0	2.3		18.0
Calcium creatinine ratio	<0.2	0.14			0.81	0.6	3.32	0.24		0.53	2.8	0.2
eGFR	>60				141							58

Working diagnoses conferred on this patient throughout his treatment course

1. Idiopathic Nephrotic Syndrome and Minimal Change Nephrotic Syndrome (After Biopsy) 

Working diagnosis at 18 months of age due to proteinuria (3+ urine dipstick), elevated UPC and failure to thrive. Lack of edema, renal insufficiency and hypertension were points against this diagnosis. A trial of steroids was given.

2. Nutritional Rickets

Working diagnosis at four and a half years of age as the child presented with bowing of legs (genu valgum) with severe stunting. Vitamin D therapy was initiated along with the steroids.

3. Hypervitaminosis D 

Presentation with vomiting, irritability, polyuria and abdominal pain, following mega doses of vitamin D.

*4. Steroid Toxicity (Iatrogenic Cushing’s Syndrome*)

High dose steroids for increased periods of time were the cause. Points favouring the diagnosis were cushingoid facies, irritability, hypertension and bloating. Symptoms resolved after gradual tapering of the steroids.

5. Steroid Resistant Nephrotic Syndrome (SRNS) 

Diagnosed due to lack of therapeutic response after six weeks (plus two + four + two weeks) of oral prednisolone (60mg/m^2^) and three pulses of intravenous methylprednisolone (500mg/m^2^). The biopsy pattern of focal segmental glomerulosclerosis (FSGS) was supportive and calcineurin inhibitor (CNI) therapy was initiated. Again, however, the lack of classical and atypical nephrotic syndrome findings such as edema, hypertension, systemic autoimmune disorder and the presence of genu valgum, all pointed to a different diagnosis.

6. Hypophosphatemic Rickets 

Diagnosed due to normal serum albumin levels, proteinuria, hypercalciuria, medullary nephrocalcinosis, vitamin D resistance and decreased TmP-GFR with no edema. Therapy with oral potassium, phosphate, angiotensin-converting enzyme inhibitors and atorvastatin was instituted. However, genetic sequencing for Dent disease, growth hormone and IGF-1 levels was not done.

## Discussion

As is evidenced, our patient has had a significant delay in the diagnosis of Dent disease, which led to an irreversible cascade of events that could have been avoided. As per a study conducted by Bhardwaj et al. in 2016 on a cohort of Indian children with Dent disease [4], the mean age of onset was 1.8 years which is consistent with our patient, while the mean age of diagnosis was 8.0 years. In our patient, a definitive diagnosis was established after around 13 years. Even though he was diagnosed as having a phenotype of hypophosphatemia, targeted interventions such as GH counselling and therapy were not implemented. Progression to kidney failure occurs usually around the third to fourth decade of life, but a much more rapid progression in the first decade is described in patients harbouring the p.Ser244Leu mutation [4], which alters the ClC5 α-helix G that interferes with dimer interface formation [5]. Even though our patient harbours this mutation, a progression to end-stage renal disease (ESRD) in the first decade was not seen. However, most male patients with Dent disease develop CKD with an estimated decline in GFR of 1.0 to 1.6 mL/min/1.73m^2^ per year [6]. In our patient, only two values were obtained, which showed a fall in eGFR of close to 10ml/min/1.73m^2^ per year over eight years.

Dent disease should be suspected in a patient with the three following criteria in the absence of other known causes of proximal tubule dysfunction [7,8].

1. Low molecular weight proteinuria (LMWP) at least five times above the upper limit of normal.

2. Hypercalciuria - Adults (age >18 years): >4.0mg calcium (0.1 mmol/kg) in 24 hours or >0.25 calcium/creatinine mg/mg (0.57 mmol/mmol) in spot urine; Children: See Table 2 for 95th percentile for urine calcium/creatinine (mg/mg) reference values [9].

**Table 2:** Calcium/creatinine (mg/mg) reference values in children (age <18 yrs)

Age (years)	95th percentile
0-1	<0.81
1-2	<0.56
2-3	<0.50
3-5	<0.41
5-7	<0.30
7-10	<0.25
10-14	<0.24
14-17	<0.24

3. At least one of the following: Nephrocalcinosis, nephrolithiasis, hematuria (macroscopic or microscopic), hypophosphatemia, CKD, glomerular filtration rate (GFR) that is below the normal limits for age, family history consistent with X-linked inheritance

A definitive diagnosis is established in a male by identification of a hemizygous pathogenic variant in either CLCN5 (Dent disease 1) or OCRL (Dent disease 2) by molecular genetic testing. Female carriers are usually heterozygous for disease-causing variants, but some may have mild symptoms like hypercalciuria, LMWP and others with nephrolithiasis and rarely CKD progressing to ESRD. These rare cases are likely due to the phenomenon of skewed X chromosome inactivation.

Poor compliance is likely responsible for poor control of analytes and is well reflected in Table 1. Persistent hypercalciuria which has led to nephrocalcinosis is evidenced by elevated urinary calcium creatinine ratio and 24-hour urinary calcium excretion. Frequent hypokalemic readings after diagnosis are attributed to the use of thiazide diuretics. However, cases of Dent disease occurring concomitantly with Bartter syndrome have been observed [10]. Hypercholesterolemia in Dent disease is not a well-known entity, yet it seems to be significant in our patient over the years, being refractory to statin therapy. Renal biopsy is not required to make a diagnosis of Dent disease but is often obtained as patients present with proteinuria, CKD and/or other renal pathologies. In a study conducted by Wang et al. [11], FSGS is reported in 83% while tubulointerstitial fibrosis is seen in 60% of patients with Dent disease. In our patient, the biopsy pattern was initially minimal change disease which changed to FSGS on a repeat biopsy. This is consistent with steroid-resistant nephrotic syndrome (SRNS) where repeat biopsies, in a study with 171 patients with SRNS found that the diagnosis changed from minimal change disease to FSGS in 55% of cases [12]. Moreover, FSGS is seen in 35% to 55% of patients, minimal change disease in 25% to 40% of patients and idiopathic mesangioproliferative glomerulonephritis in 10% to 15% of patients with SRNS [13].

It has been established that there is no genotype-phenotype correlation in this disease spectrum and varied clinical presentations are the norm. Moreover, the phenotypic differences across the globe are likely due to a combination of environmental and dietary factors as well as delay in diagnosis and the effect of modifier genes. In addition to the urinary findings, in the Indian study conducted by Bhardwaj et al., the following have been identified as early or presenting symptoms: short stature attributed to rickets (100.0%), polyuria/polydipsia (88.9%) and night blindness (66.7%) [5]. Polyuria and polydipsia are attributed to secondary nephrogenic diabetes insipidus and may be mediated by the downregulation of aquaporin-2 [14]. Rickets in the same study was refractory to vitamin D therapy in all subjects (as was in our patient) as compared to a study by Wrong et al. [15], in which vitamin D therapy was beneficial. Furthermore, short stature is more pronounced in Dent disease type 2 as compared to type 1. Night blindness is attributed to urinary loss of retinol-binding protein. In a European study conducted by Blanchard et al. [6], however, rickets was the presenting feature in only (19%) of patients. Presenting features in this group in addition to the urinary findings were incomplete (73%) and complete (11%) Fanconi syndrome, nephrocalcinosis (42%) and nephrolithiasis (32%). The appearance of lower extremity bowing or deformities within the first two years of life (corresponds to weight-bearing) and with normal serum levels of vitamin D and calcium, should alert the pediatrician towards a possibility of a phenotypic spectrum of X-linked hypophosphatemia, and possibly Dent disease. An algorithmic approach [16], published in the journal 'International Urology and Nephrology' is an option to consider to reach a prompt diagnosis. 

**Figure 1:**
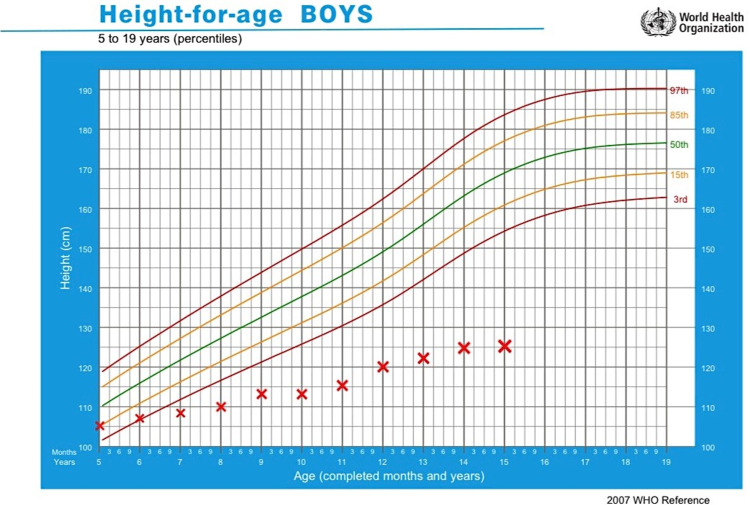
World Health Organization (WHO) height-for-age graphical plot of our patient from five years of age

The primary goals of treatment are to decrease hypercalciuria, prevent kidney stones and nephrocalcinosis and delay the progression to ESRD. The use of thiazide diuretics at doses greater than 0.4mg/kg/day has decreased urinary calcium excretion by more than 40% [17], but frequent occurrence of side effects, especially hypokalemia limit its use. There is no evidence of using potassium-sparing diuretics in Dent disease to reduce the occurrence of hypokalemia. Data on angiotensin-converting enzyme (ACE) inhibitors and angiotensin receptor blocker (ARB) use have been inconclusive. However, a significant reduction in the urinary albumin creatinine ratio was observed using angiotensin-centred therapies in 54% of children after a median of 1.7 (range 0.3 to 8.5) years of treatment in a recent study by Deng et al. on a cohort of 31 children with Dent disease [18]. Renal replacement therapy including renal transplant is indicated for patients with ESRD. Prevention of secondary complications such as bone disease or rickets is of utmost importance in achieving the target height and the lack of which can be seen in our patient. A trial of vitamin D and calcium supplements can be considered as some patients are responsive. Frequent oral administration of phosphate can minimize the bowing of long bones during growth. Growth hormone (GH) therapy is now considered in patients who are stunted with low serum GH and IGF-1 levels. In a study by Sheffer et al., treatment of short stature in subjects aged around 10 years with GH doses typical for GH deficiency (0.3mg/kg/week) resulted in a marked improvement in IGF-1 levels to within the normal range in the first four months and improvement in growth rate was noted by nine months [19]. In our patient, the family was never counselled regarding the prospect of GH therapy and GH levels were never estimated, even though his height was below the third centile for his age, throughout. At his current age, the patient did not qualify for GH therapy. A height-for-age graphical plot of our patient from age five years is shown in Figure 1 [20]. Early identification of the GH deficiency state and correction of the same at an earlier stage should have been the norm. Complications such as dental abscesses related to hypophosphatemia can be prevented with good oral hygiene, regular flossing and dental care. There are currently no guidelines regarding screening for cardiovascular complications in Dent disease given the persistent hypercholesterolemia in our patient.

## Conclusions

Dent disease type 1 is an X-linked tubulopathy mainly caused by inactivating mutations in the CLCN5 gene. Due to similarities in presentation of the disease to other commonly seen pediatric conditions (SRNS, nutritional rickets, renal tubular acidosis, etc.) and the fact that the disease has variable phenotypes and allelic heterogeneity, a delay in diagnosis is often encountered. A high index of suspicion and early molecular testing is ideal and prevents the hazards of overtreatment, multiple invasive procedures and early targeted interventions such as growth hormone, phosphate and thiazide/ACE inhibitor therapy can be considered. Renal failure usually occurs in the third to fourth decade but may occur in the first decade of life with the most commonly described CLCN5 mutation (Ser244Leu). Guidelines regarding the management of hypercholesterolemia in Dent disease are sparse and additional research regarding this pathogenesis is warranted. Genetic counselling, patient education and screening of family members especially males, are pivotal and is recommended in all cases. 
